# Sexual homicides in South Africa: A national cross-sectional epidemiological study of adult women and children

**DOI:** 10.1371/journal.pone.0186432

**Published:** 2017-10-17

**Authors:** Naeemah Abrahams, Shanaaz Mathews, Carl Lombard, Lorna J. Martin, Rachel Jewkes

**Affiliations:** 1 Gender & Health Research Unit, South African Medical Research Council, Cape Town & Pretoria, South Africa; 2 Children’s Institute, Faculty of Health Sciences, University of Cape Town, Cape Town, South Africa; 3 Biostatics Unit, South African Medical Research Council, Cape Town, South Africa; 4 Forensic Pathology Services, Western Cape/ Forensic Medicine & Toxicology, University of Cape Town, Cape Town, South Africa; Stellenbosch University, SOUTH AFRICA

## Abstract

Sexual homicides of women and children are extreme forms of violence against women and children and are located within the broader context of gender inequality and social norms that condone sexual violence and gender based violence against women and children. The aim of this study is to describe the prevalence and characteristics of sexual homicide of women and children nationally in South Africa.

**Methods:**

We conducted a retrospective national mortuary based study to identify all adult female homicides (18 years and older) and all child homicides (boys and girls < 18 years) in 2009 in a randomly selected, proportionate sample of mortuaries. Victim, perpetrator and crime data were collected in three processes: from the mortuary register, the autopsy report and from police with the identification of sexual homicides validated across the data collection processes.

**Findings:**

Among the 2670 (95% CI: 2311–2979) adult women killed in 2009, 494 (95% CI: 406–574) were identified as sexual homicides which was 19.8% (95% CI: 17.6–22.0) of all adult female homicides and among 1277 (95% CI: 1091–1462) children killed in SA, sexual homicides were found in 104 (95% CI: 77–132) of the child homicides which was 8.7% (95% CI: 10.9–11.2%) of these murders. Strangulation was the most common cause of death for both children and adult females. A distinct age and sex pattern was found among children with only 1% boy child death identified as a sexual homicide and 92% of all the child sexual homicides were among girls. Strangulation was the most common manner of death among children (35.5%) and perpetrators were seldom strangers. However, no difference in the proportion of convictions between the sexual homicides and non-sexual homicides were found for both adult females and children.

**Conclusion:**

Rape homicide is not a rare event in South Africa, with one in five female homicides and nearly one in ten child homicides identified with an associated sexual crime. These high prevalences are amongst the highest levels reported in the literature with our study among the few reporting on the epidemiology of child sexual homicide. Reducing mortality is an important policy goal for South Africa and for the rest of the world and the prevention of female and child homicide is an important part of attaining this goal.

## Introduction

Sexual homicide of women is a distinct form of gender-based violence that combines two of the most extreme forms of violence against women. It is a violation that garners exceptional community outrage, as seen in the global response in 2013 to the gang rape and murder of Jyoti Singh Pandey in India and the brutal rape and murder of Anene Booysen in South Africa[1]. Sexual homicide has been described as a rare event in many countries such as United States (US) where under 1% of all homicides (male and female) are identified as sexual homicides [2] and in the United Kingdom (UK) where 3.7% of those found guilty of homicide included a sexual violence component[3]. However a recent study from Alaska found 17% of all homicides to be sexual homicides [4]. The high estimate in the Alaskan study may be largely due to forensic pathologists working closely with forensic nurse specialists who assisted with the identification of sexual assaults during postmortem examinations. Whilst the Alaskan estimate was much higher than standard estimates from the USA, it was very similar to that reported in a South African study on female homicide where, in a national sample, 16.3% of all the female homicides in 1999 were identified as sexual homicides [5]. Thus, in these two settings it is not rare events. It was also very similar to the proportion of sexual assault found in a 10-year study (2000–2009) of all female homicides seen in a Forensic Medical Laboratory (mortuary) in the Western Cape, South Africa which was 21.1% [6].

There is also very little epidemiological data on sexual homicides of children. Available data are from forensic laboratories in develop settings, such as the Schmidt and Madea report on three child sexual homicides identified over a period of 5 years (1992–1996) from a German University Forensic department database [7]. This article describes the forensic aspects of the injuries. The 8-year follow-up study from Alaska retrieved data from forensic nursing reports and found 20% of the 8-year sample were children under 18 years. In South Africa child sexual homicides were reported as part of a study of femicides in the Cape Town region, and one in three (32.5%) of all the female child homicides were identified as sexual homicides. The paper described 30 child sexual homicides cases over a period of 10 years [6].

Sexual violence is a common feature in the lives of many adult women and children in South Africa as shown in population studies [8,9] and sexual homicides are therefore located within the broader context of gender inequality and the underlying system of patriarchy which drives violence against women and children [10]. Research was conducted on homicides in 1999 in South Africa in a national study of female homicide [11]. In order to examine trends in homicide, similar research was conducted ten years later [12]. In this paper, we describe the prevalence of sexual homicide of adult women and male and female children in 2009 in South Africa, forensic aspects of the cases, and social and demographic characteristics of the victims and perpetrators. In drafting this paper, we acknowledge that in the literature there are differences in use of the terms rape, sexual violence and sexual assault. In this study, we define sexual homicide as cases where the post-mortem report included a statement that sexual assault was suspected.

## Methods

Our study combines data extraction from health and police records and interviews with police officers. The South African Inquest Act of 1959 require post mortem examinations on all unnatural deaths and this allowed us to collect data on female and child homicide cases that were presented to medical legal laboratories between 1 January 2009 and 31 December 2009. We conducted a cross-sectional national retrospective mortuary based study of adult female and child homicides using a similar study design as for the 1999 female homicide study [11]. Our sampling frame included all operating mortuaries in South Africa in 2009 and we stratified the mortuaries into three strata based on the number of autopsies performed per year. The strata were: mortuaries that perform less than 500 autopsies per year (81 mortuaries): mortuaries that perform between 500–1499 autopsies per year (33 mortuaries) and the largest strata were mortuaries that perform more than 1500 autopsies per year (8 mortuaries). We drew a random sample of 38 mortuaries based on proportional allocation (see Table 1 for sampling fraction). Our sample size calculation was based on the national estimates from the 1999 study.

**Table 1:** Sampling strata and sampling fraction of sampling frame.

Mortuary strata	Number of mortuaries	Sampling fraction
**Small:** < 500 autopsies performed per year	81	24.7%
**Medium:** 500–1499 autopsies performed per year	33	39.4%
**Large**: > 1499 autopsies performed per year	8	55.5%

Our data collection process followed a series of steps: first identification of homicide cases from the mortuary death registers, followed by verification of the homicides cases from the data in the post mortem report, and finally interviews with the police investigator for each of the verified cases. Confirmation that the case was a homicide happened at each step, and we extracted the data at each step onto a data capture sheet. Data extracted from the mortuary register included initial cause of death recorded, demographic details of victim (age, sex), identity of police station where case was reported and the case number. The police station and case number were critical to identify and contact the police investigating officers. Data extracted from the post mortem report included injury and pathology data: verification of the cause of death, demographic information and evidence of sexual assault at time of murder and pregnancy status. As for the 1999 study, we defined sexual assault as cases where there was evidence of a sexual component that was not limited to penetration of the genitalia or anus by a penis. This is similar to the definition by Meloy where sexual homicide is defined as a homicide with a ‘sexual element’ [13] (p 2). In our study, sexual homicide cases were those where one or more of the following were recorded in the post mortem report: a statement that sexual assault was suspected; reporting of genital injuries; reporting that body found with clothes/underwear dislodged or removed; the use of sexual assault evidence collection kit or a vaginal swab or sample of pubic hair taken. We also asked the investigating officer at the interview about evidence from the investigation that indicated sexual assault as part of the homicide. During the data collection process if we received information which contradicted the suggestions that a case was a homicide or a sexual homicide we reviewed the case and dropped those from the sample (or this analysis) that on balance did not fit the categorization.

Further information collected from the police included more victim and perpetrator information (where known) such as socio-demographic details of the victim and perpetrator, the relationship between victim and perpetrator (intimate partner, acquaintance, stranger etc.), victim information (i.e. more than one victim,) details of the murder (scene of crime, weapon used), perpetrator information (number of perpetrators), the investigation and the outcome of the investigation (charged, convicted, length of sentence). We present available sentencing information, but this was not present for most of the children.

The analysis is based on the sexual homicide cases. Information to determine if there was a sexual homicide was not available for 5.4% (n = 66) of the child murders and in 5.9% (n = 157) of the adult women murdered. Data was analysed using STATA release 13[14]. Weighted estimates are presented, as the analysis took into account the study design and allocation of the different sampling weights. We present analysis for adult women (18 years and older) and male and female children (<18 years) separately, and provide prevalence estimates with 95% Confidence Intervals (CI) using standard methods for estimating confidence intervals for complex multi-stage sample surveys (Taylor linearization).

We calculated sexual homicide rate for female and male children 0–17 years and for adult women for 18 years and older. We also calculated the sexual homicide rate for females 14 years and older in order to compare with the 1999 study. We used the mid-year population estimates from Statistics South Africa for 2009 which were extrapolated from the 2001 census [15]. These population data are commonly used for government and administrative purposes and were adjusted for population growth and undercount. We also calculated the rate per sexual offences reported to police using the data for the 2009/2010 year (the crime reporting year is from April 2009 to March 2010)[16]. The crime reporting does not disaggregate data for women and children or for male and female and we combined the number of sexual homicides for children and women and used the total figure of 66 992 sexual offences reported as the denominator.

Ethics approval for the study was granted by the South African Medical Research Council Ethics Committee (Protocol ID: EC09-021) and access to the state mortuaries was granted by the National and Provincial Department of Health while access to police stations and police investigating offers were granted by the National and Provincial Police Service and the National Prosecuting Authority. The Police investigating officers were required to provide written informed consent.

## Results

Among the 2670 (95% CI: 2311–2979) adult women killed in 2009, 494 (95% CI: 406–574) were identified as sexual homicides which was 19.8% (95% CI: 17.6–22.0) of all adult female homicides. Among the 1277 (95% CI: 1091–1462) children killed in South Africa, sexual assault was found in 104 (95% CI: 77–132) of the child homicides which was 8.7% (95% CI: 10.9–11.2%) of these homicides. We found an adult female sexual homicide rate of 3.05/100 000 women 18 years and older and the child sexual homicides rate of 0.56/100 000 children <18 yrs (Table 2). In children, we found a higher rate among female children (1.03/100 000 girl children) than male children (0.08/100 000 boy children). The combined rate of women and children per 1000 sexual offences reported to police in the 2009/2010 reporting year was 8.92/1000 (95% CI: 6.9–10.4).

**Table 2:** Population rates for sexual homicides of children and adult females (weighted estimates).

Sexual Homicide	Population	N (95% CI)	Rate per 100 000 (95% CI)
Adult females 18+ years	16 185 293	494 (406–574)	3.05 (2.50–3.54)
Females 14 + years	15 360 904	455 (306–605)	2.5 (1.7–3.3)
			
All children 0–17 years	18 529 472	104 (77–132)	0.56 (0.41–0.71)
Female children 0–17 years	9 266 507	96 (70–122)	1.03 (.75–1.31)
Male children 0–17 years	9 262 965	8 (2–14)	0.08 (0.02–0.16)

Table 3 presents the socio demographic and crime data of sexual homicides for adult women and children. Although boy children represented a large proportion of all the children murdered (64.2%) they represented only 8% of the sexual homicide victims. The youngest child in the sexual homicide group was a girl of 1 year 8 months old and the youngest boy was 3 years old. The age profile of the child victims showed older children were at greater risk with the 13–18 year group representing more than half (52.3%) of the sexual homicide children. This is also evidence in the difference in mean age between the two groups. The age profile of adult women showed similar proportions among the sexual homicide and the non-sexual homicide group (*p* = 0.54).

**Table 3:** Frequencies of socio-demographic and pathology outcomes for child and adult women sexual homicides: (weighted estimates).

	Adult women (18 + yrs) n (95% CI)					Children (0–17 yrs) n (95% CI)			
	All adult women Homicides	Sexual homicides		Non-Sexual Homicides	*P* value	All child homicides)	Sexual homicides	Non-Sexual Homicides	*P* value
	n = 2645 (2311–2979)	n = 494 (406–574)		n = 2019 (1758–2281)		n = 1277 (1091–1462)	n = 104 (77–132)	n = 1081 (903–1260)	
		19.8% (17.6–22.0)		80.2%(77.9.82.3)			8.7% (6.7–11.1)	91.3% (88.8.93.3)	
**Sex of child victims**									
Boy						64.2 (60.2–68.2)	8.0 (3.7,16.5)	29.3 (25.2,33.6)	<0.00
Girl						(31.8–39.5)	92.0 (83.5–96.3)	70.7 (66.4,74.8)	
**Victim age**									
Mean age	38.2 (37.3–39.1)	38.6 (36.6–40.6)		37.86 (26.8–38.9)		9.1 (7.9–10.3)	11.0 (9.7–12.4)	9.3 (7.4–10.9)	
Standard Deviation	15.7	16.4		15.4		7.4	5.2	7.6	
Median age	35	35.8		34.5		11.6	12.4	12.0	
**Victim age categories**									
<5 years						41.1 (34.1–48.5)	15.3 (8.6–25.8)	42.5 (34.4–50.9)	0.10
5-<13						10.1 (8.5–12.1)	32.3 (21.9–44.8)	7.4 (5.5–9.8)	
13-<18						48.8 (41.2–56.4)	52.3 (40.2–64.2)	50.1 (41.2–59.0)	
18-<30	35.5 (32.6–38.5)	35.7 (31.7–39.9)		35.7 (32.5–39.1)	0.54				
30-<45	36.8 (33.4–40.3)	35.4 (31.7–39.2)		37.5 (33.5–41.5)					
45-<60	15.9 (14.0–17.9)	17.6 (14.1–21.9)		15.3 (13.3–17.7)					
60+	11.7 (9.9–13.7)	11.3 (8.0–15.7)		11.3 (9.2–13.8)					
*Victim employment									
Employed	24.5 (21.9–27.2)	23.1 (18.8–28.0)		26.0 (23.6–28.6)	0.40				
Not employed	53.0 (48.8–57.2)	54.2 (49.0–59.4)		51.3 (47.2–55.4)					
Employment status unknown	22.5 (19.4–25.9)	22.7 (19.1–26.6)		22.7 (19.4–26.3)					
**Crime happened in**									
Urban area	50.4 (44.5–56.2)	52.0 (42.1–61.8)		50.8 (45.3–56.3)	0.73	51.0 (42.8–59.1)	49.2 (38.9–59.5)	50.9 (41.7–59.9)	0.76
Small town/rural	49.6 (43.8–55.5)	48.0 (38.2–57.9)		49.2 (43.7–54.7)		49.0 (41.0–57.2)	50.8 (40.5–61.1)	49.2 (40.1–58.2)	
**Crime scene**									
Home	62.4 (60.1–64.6)	46.0 (38.9–53.2)		67.2 (64.5–69.8)	<0.00	44.1 (40.0–48.4)	47.9 (35.6–60.5)	44.5 (40.0–49.0)	0.38
Public space	31.0 (29.1–33.0)	47.7 (40.9–54.5)		26.7 (24.9–28.7)		44.7 (40.5–48.9)	43.2 (30.9–56.4)	44.4 (40.0–48.8)	
Other	3.1 (2.3–4.2)	4.0 (2.4–6.6)		2.4 (1.7–3.6)		7.2 (5.4–9.7)	2.5 (.05–11.1)	7.2 (5.1–9.9)	
Unknown	3.5 (2.9–4.2)	2.4 (1.2–4.5)		3.7 (2.9–4.6)		4.0 (2.9–5.4)	6.4 (2.8–13.9)	4.0 (2.8–5.6)	
**Multiple victims**									
Yes	13.1 (11.7–14.7)	11.6 (8.9–14.8)		13.6 (11.7–15.7)	0.29	18.9 (15.7–22.5)	15.6 (6.8–31.7)	19.0 (15.6–22.7)	0.61
No	86.9 (85.3–88.3)	88.5 (85.2–91.1)		86.4 (84.3–88.3)		81.1 (77.5–84.3)	84.4 (68.2–93.2)	81.0 (77.3–84.2)	
**Victim was pregnant**									
Yes	5.2 (3.7–7.1)	6.9 (4.1–11.6)		4.4 (3.5–5.5)	0.02	2.4 (1.2–4.8)	0	3.1 (1.5–6.4)	0.23
No	94.8 (92.9–96.3)	93.1 (88.4–95.9)		95.6 (94.5–96.5)		97.6 (95.2–98.8)	100	96.9 (93.6–98.5)	
**Cause of death**									
Gunshot	23.1 (20.0–26.6)	8.9 (6.1–12.8)		27.2 (23.6–30.9)	<0.00	14.1 (11.6–17.0)	0	16.4 (13.5–19.8)	<0.00
Stabbed	30.0 (26.3–34.0)	24.9 (20.7–29.5)		32.6 (28.5–37.0)		23.3 (18.1–29.3)	21.2 (12.9–32.8)	24.3 (18.6–31.2)	
Blunt	19.1 (16.3–22.2)	25.5 (20.8–30.9)		17.9 (14.9–21.3)		16.5 (13.6–19.8)	20.1 (10.4–25.5)	16.8 (13.5–20.8)	
Strangled	7.9 (6.8–9.0)	24.5 (20.1–29.5)		3.6 (2.8–4.7)		6.5 (5.2–8.0)	35.5 (24.3–48.5)	4.0 (2.9–5.4)	
Asphyxiated	0.8 (0.4–1.3)	2.2(1.1–4.5)		0.3 (0.1–1.1)		2.8 (1.8–4.4)	6.5 (2.5–15.8)	2.7 (1.7–4.2)	
Multiple injuries	4.8 (3.7–6.2)	3.4 (2.0–5.7)		4.1 (3.1–5.3)		4.6 (3.1–6.6)	3.9 (1.4–10.6)	3.5 (2.3–5.4)	
Undetermined	5.1 (4.0–6.5)	6.2 (4.6–8.4)		4.8 (3.4–6.7)		3.9 (2.8–5.4)	11.1 (6.0–19.7)	3.5 (2.4–5.1)	
Other **	8.8 (7.2–10.7)	4.4 (2.8–6.8)		9.6 (7.7–11.8)		28.1 (22.6–34.4)	1.7 (0.3–9.6)	28.6 (22.4–36.2)	

*employment data collected for children from the age 14 years

** abandoned neonates included in this group

There were similar levels of sexual homicides in urban and rural settings for both children (*p* = 0.76) and adult women (*p* = 0.73). The crime scene data showed that among the adult sexual homicides, similar proportions of the crime happened in homes and in public spaces, while non-sexual homicides were more likely to happen in the home (67.2% vs 26.7%). For children, there was no difference in the crime scene between the sexual and non-sexual homicides (*p* = 0.38). Multiple victim crime was also not a common feature of sexual homicides for both children and adult women. Low levels of pregnancy were reported among all of the women (5.2%) and children (2.4%) killed, but when the proportion of sexual homicides were explored among those who were pregnant, high levels were found for both the adult women (27.2%) and children (19%) (data not shown in Table 3). Strangulation was only ranked fourth as the cause of death among all the homicides for adults (7.9%) and 5^th^ for all children (6.5%), but higher proportions were found among the sexual homicide cases of adult women (24.5%). Strangulation was the main cause of death among the child sexual homicides cases (35.5%) with much lower proportion reported among the non-sexual homicides (4.0%). Gun injuries were not common among the sexual homicides for both adult women (8.9%) and children (0%).

Table 4 shows a comparison of perpetrator characteristics by whether homicides were sexual or non-sexual. Higher levels of unidentified perpetrators were found among the sexual homicide cases compared with non-sexual homicide cases for both adult women (35.5% vs 22.9%) and children (29.5% vs 20.6%) but this difference was only significant for adults (*p* = <0.00). Although intimate partners perpetrated 42.8% of all adult female homicides (18 years and older), only 22.5% of the sexual homicides were committed by an intimate partner with acquaintances perpetrating more than a quarter of these crimes (27.6%). Acquaintances were also the most common perpetrators (37.5%) of child sexual homicides while family members were the most common perpetrators of the non-sexual homicide cases (42.6%). The group we describe as ‘other perpetrators’ include gang members, co-workers, teachers, employees, and service providers such as police. More unknown perpetrators were found in the children sexual homicide group compared with the non-sexual homicide group (11.3% vs 3.1%). A significant difference in the mean age of the perpetrators was found for adult female sexual homicides, with perpetrators who committed sexual homicide younger than those who committed non-sexual homicides (30.9 years vs 33.4 years). In contrast, among children the perpetrators of child sexual homicide were older than the perpetrators who committed non-sexual homicide (27.8 years vs 25.1 years).

**Table 4:** Frequencies of perpetrator characteristics for child and adult women sexual homicides (weighted estimates).

	Adult women (18 + yrs) n (95% CI)				Children (0–17 yrs) n (95% CI)			
	All adult women Homicides	Sexual homicides	Non-Sexual Homicides	*P* value	All child homicides	Sexual homicides	Non-Sexual Homicides	*P* value
	n = 2645 (2311–2979)	n = 494 (406–574)	n = 2019 (1758–2281)		n = 1277 (1091–1462)	n = 104 (77–132)	n = 1081 (903–1260)	
		19.8% (17.6–22.0)	80.2%(77.9.82.3)			8.7% (6.7–11.1)	91.3% (88.8.93.3)	
**Perpetrator was identified**								
Identified	60.5 (57.0–63.9)	48.6 (42.0–55.2)	64.2 (60.4–67.8)	<0.00	58.9 (53.6–64.0)	58.1 (44.5–70.6)	59.8 (54.1–65.3)	0.19
Suspected only	13.4 (12.3–14.8)	15.9 (13.2–19.2)	12.9 (11.6–14.4)		18.9 (15.2–23.2)	12.4 (7.2–20.7)	19.6 (15.4–24.6)	
Remained unidentified	26.1 (23.0–29.5)	35.5 (29.1–42.3)	22.9 (19.8–26.3)		22.17 (18.1–26.9)	29.5 (17.2–45.7)	20.6 (16.6–25.4)	
**Relationship with victim**								
Intimate partner	42.8 (39.6–46.0)	22.5(18.3–27.4)	48.2 (44.5–52.0)	<0.00	3.6 (2.4–5.3)	1.8 (0.3–9.8)	3.2 (2.0–5.3)	<0.00
Family member	5.6 (4.3–7.10	4.4 (3.0–6.4)	6.0 (4.4–8.1)		41.8 (36.6–49.1)	27.1 (19.7–35.9)	42.6 (36.5–48.8)	
Acquaintance	17.8 (15.8–20.0)	27.6 (22.6–33.1)	15.7 (13.9–17.6)		33.4 (27.8–39.5)	37.5 (28.7–47.1)	34.2 (28.4–40.5)	
Stranger	7.6 (6.1–9.4)	9.9 (6.5–14.7)	7.3 (5.8–9.3)		4.5 (3.5–5.9)	6.7 (2.6–15.9)	4.7 (3.5–6.1)	
Other	0.9 (0.6–1.5)	1.8 (0.8–3.8)	1.8 (0.5–1.2)		2.7 (10.0–15.9)	15.7 (9.3–25.4)	12.3 (9.5–15.8)	
Unknown	25.1 (21.7–28.8)	33.8 (27.4–40.9)	22.0 (18.7–25.7)		3.9 (3.0–5.0)	11.3 (5.7–21.2)	3.1 (2.2–4.3)	
**Mean perpetrator age**								
Mean age	32.9 (32.2–33.5)	30.9 (29.4–32.4)	33.4 (32.5–34.3)	0.02	25.4 (24.3–26.4)	27.8 (25.2–30.4)	25.1 (23.9–26.1)	0.08
**Perpetrator employed**								
Yes	32.0 (28.6–35.6)	23.0 (17-8-29.1)	34.4 (31.0–38.1)	<0.00	17.8 (15.5–20.8)	23.1 (16.4–31.4)	16.5 (13.2–20.4)	0.30
No	33.1 (30.2–36.2)	35.7 (29.9–42.0)	33.5 (30.2–36.9)		48.2 (42.9–53.6)	50.4 (39.0–61.7)	49.3 (43.5–55.0)	
Unknown	34.9 (30.8–39.3)	41.3 (35.2–47.7)	32.1 (27.7–36.9)		34.0 (29.8–38.4)	26.6 (15.1–42.4)	34.2 (29.7–39.2)	
**Multiple perpetrators**								
Yes	13.5 (12.1–15.0)	17.4 (14.3–21.0)	12.7 (11.0–14.6)	<0.00	17.3 (12.9–22.8)	6.0 (2.4–14.4)	19.0 (14.3–24.9)	0.03
No	61.9 (58.2–65.4)	47.7 (20.1–55.4)	66.2 (62.5–69.8)		60.6 (56.5–64.6)	68.7 (55.7–79.3)	59.6 (54.7–64.3)	
Unknown	24.7 (21.2–28.6)	34.9 (28.8–41.7)	21.1 (17.5–25.3)		22.2 (17.4–27.8)	25.3 (15.0–39.3)	21.4 (16.3–27.6)	
*Convicted								
Yes	26.5 (23.2–30.1)	28.3 (22.1–35.6)	26.7 (23.5–30.1)	0.59	38.8 (29.3–49.1)	46.5 (19.0–76.3)	33.3 (25.9–41.5)	0.44
No	73.5 (69.9–76.6)	71.7 (64.5–77.9)	73.4 (69.9–76.5)		61.2 (50.8–70.6)	53.5 (23.8–81.0)	66.7 (58.5–74.1)	
*Length of sentence given								
< 5 years	6.5 (4.4–9.5)	6.2 (1.9–18.6)	5.5 (3.4–8.7)	<0.00	15.4 (9.6–23.9)	0	19.8 (12.7–29.5)	<0.00
5-<10 years	20.9 (16.0–27.0)	10.9 (5.0–22.0)	23.9 (18.2–30.7)		26.1 (20.5–32.7)	0	32.2 (25.1–40.2)	
10-< 15 years	28.0 (22.8–33.8)	12.4 (5.5–25.8)	32.3 (25.9–39.5)		19.1 (13.6–26.0)	17.3 (6.7–37.9)	20.1 (13.6–28.6)	
15+ years	44.5 (38.9–50.4)	70.5 (59.2–79.8)	38.3 (30.9–46.3)		39.4 (30.5–49.1)	82.7 (62.1–93.3)	27.9 (19.4–38.4)	

*convict and sentencing information not available for girl children 14 years and younger

About one third of the perpetrators of the adult victims were employed and for another third the employment status remained unknown. A significant difference (*p* = <0.00) between the proportions of employed perpetrators was found with lower levels of employment among adult sexual homicide perpetrators compared with non-sexual homicide perpetrators (23.0% vs 34.4%). Very little differences were found in the employment status of perpetrators among the children (*p* = 0.30). The involvement of multiple perpetrators was more common among adult sexual homicides cases (17.4%) than adult non-sexual homicide cases (12.7%) but this was reversed for children with multiple perpetrators not common (6% vs 19%).

There was no difference in the proportion of convictions among the sexual homicides and non-sexual homicide victims among adult females (*p* = 0.59) while for children the convictions among the sexual homicide cases were higher than the non-sexual homicide cases (46.5% vs 33.3%) but this difference was not significant (*p* = 0.44). The data on the length of sentencing was only available for adult women and for girl victims older than 14 years. Perpetrators of sexual homicides were more likely to receive longer sentences (15 plus years) for both adult women and children among the sexual homicides compared to the non-sexual homicides.

## Discussion

Our study showed sexual homicides are not rare events in South Africa, with nearly 500 adult female cases in 2009. Indeed, the sexual homicide rate for adult women (3.05/100 000) is similar to the global overall female homicide rate, reported to be between 3–4 per 100 000 females [17]. The proportion of all cases of female homicide among women aged 14 and over that were sexual homicides was 19%, which was higher than that reported in the study of deaths in 1999, where the proportion was 16% [5]. However, it is similar to the 10-year study at a single large mortuary in the Western Cape, where it was reported to be 19.9% among all female homicides [6]. Our finding is similar to that of the Alaskan study (17%).

Our study showed nearly one in ten of the child homicides (8.7%) was a sexual homicide. We could not find similar rates for children in the research literature and this could be one of the first epidemiological studies of sexual homicides among children in developing settings. Older children in 13-18-year group were most at risk and a third of the sexual homicides were among the 5–13 -year group which is an age group in which only 10% of all child homicides occurred. We expected the majority of the child sexual homicides to be in the older teenage group (i.e. 13 years and older) and this is what we found. However, having evidence of sexual violence among one in three of the 5-13-year-old children points to the vulnerability of younger children. We also calculated the sexual homicide fatality rate per sexual offence reported and found 9 sexual homicides for every 1000 sexual offences reported to police. The absence of disaggregated police data prevented us from calculating a separate adult female and child rate. Similar analysis of fatality rates was not found in the literature and comparison could not be made.

Our study confirms girl children are by far at greater risk of sexual homicide than boy children. This sex pattern is resonant of the broader gender differences in experiences of violence, for example in the proportion of male and female reporting acts of sexual violence to the police [18]. It is possible that the presence of a sexual crime was sometimes missed among the boy children killed because it is not expected. We also found and we had limited information from the police interviews for the boy victims and much of the information remained unknown.

Our study showed strangulation was among the most common manner of death for the sexual homicide crimes for adult females and by far the most common for children. This information is important for investigative purposes as it can guide forensic and police inquiry. This manner of death has consistently been reported in all studies where pathology findings have been reported [5,6,19,20] and have been reported in non-fatal rape research [21]. This manner of death also points to the possibility that the primary motive may have been rape as strangulation are used to induce unconsciousness.

We showed that the most common perpetrators for the crime against children were people known to them (family members (27.1%) and acquaintances (37.5%). This is similar to findings from studies on violence against children which often occur in the context of domestic and family violence [22]. However, the findings from a systematic review on sexual homicides of children identifies strangers as the most common perpetrators [23]. Indeed, strangers were not common in both the adult females and the children homicides in our study. The review also indicated that abduction was a common operandi in sexual homicides of children [23] but we did not have similar detail on the crime in our study. The investigation of these crimes is known to be difficult and the large group of missing data on the perpetrators attest to this.

The low rate of convictions also points to cases not always well investigated with only 28% of adult women sexual homicides resulting in a conviction but a higher proportion of conviction among children cases (46.5%). This implies the majority of these crimes remained unsolved. Poor investigation of crimes such as rape have been reported in South Africa [24]. Furthermore, the use of forensic crime technology, such as DNA evidence, which is available in South Africa, is obviously not being used effectively in the identification of perpetrators [25]. During the data collection, we as researchers often felt helpless when we encountered evidence of poor investigation documented in the police files in particular, child homicide cases where child abuse was involved. The clear lack of co-ordination among health, police and social services often compromised the management and investigative outcomes of these cases. In response a Child Death Review pilot project was started in two Provinces (Western Cape and Kwa-Zula Natal) in South Africa to improve the investigation but more importantly to strengthen response systems and preventing future child deaths [26].

Our study has limitations. We identified sexual homicides from both medical forensic and crime information and for most cases a rape was clearly obvious such as injuries or other crime information. However, since it is known that rape is often overlooked we included cases where a rape kit was used to collect evidence by a forensic team. This may not be considered clear evidence of a rape crime, but the study team discussed this at length and we were guided by the practice in South Africa where a rape kit would only be used if a rape is strongly suspected. Similarly, we included cases if nudity and disarray clothing were found (underwear removed or around ankles) as these are strongly suggestive of a sexual assault among homicide and non-homicide victims [27]. Given the above it is possible that we included cases that were not rape homicides. Alternatively, we may have missed rape homicide cases that did not fit our inclusion criteria as this crime is not always detected. We erred on being conservative and discussed all cases where information was not very clear i.e. the only information was the use of a rape kit and no further validation from the investigating officer. Similarly, we used the full autopsy report to find further injury information, and additional crime scene information when uncertain if a case was a homicide (a body may have been found on the side of a road and we had to exclude motor vehicle accident injuries). Perpetrator data availability was also a limitation. One in five of the cases had no perpetrator information. We also did not have details of the circumstances of all the deaths as police data was limited and we do not know if the homicide crime was planned or whether it was an impulsive act during a sexual act. We have no information on whether any of the cases were part of a serial sexual crime, to which literature on sexual homicides often alludes [2,13].

In our previous paper we suggested protocols be developed to ensure routine screening and investigation of all female homicides for sexual crimes and proposed the inclusion of a prescribed proforma document [11]. To our knowledge this has not happened. We continue with this call for all female and child murders to have focused investigations by the police and the forensic pathologist for evidence of a sexual crime. In addition, this research has enabled us to address some of the gaps in the current literature on sexual homicide of adult women and children including the bias towards data from high income countries (1).

## Conclusion

Our research shows that sexual homicide is not a rare event in South Africa with one in five female homicides and nearly one in ten child homicides identified, as such this is among the highest reported in the literature. The study also shows the proportion of sexual homicides among all female homicides have increased between 1999 and 2009. Sexual child homicides also show different risk patterns for boy and girl children. Reducing mortality is an important policy goal for South Africa and for the rest of the world and the prevention of female and child homicides is an important part of attaining this goal.
